# *Drosophila melanogaster* as an alternative model organism in nutrigenomics

**DOI:** 10.1186/s12263-019-0641-y

**Published:** 2019-05-06

**Authors:** Nieves Baenas, Anika E. Wagner

**Affiliations:** 1 Institute of Nutritional Medicine, University of Lübeck, Ratzeburger Allee 160, 23538 Lübeck, Germany; 2 Institute of Nutritional Sciences, Justus-Liebig-University, Wilhelmstrasse 20, 35392 Giessen, Germany

**Keywords:** *Drosophila melanogaster*, Nutrigenomics, Diet, Model organism

## Abstract

Nutrigenomics explains the interaction between the genome, the proteome, the epigenome, the metabolome, and the microbiome with the nutritional environment of an organism. It is therefore situated at the interface between an organism’s health, its diet, and the genome.

The diet and/or specific dietary compounds are able to affect not only the gene expression patterns, but also the epigenetic mechanisms as well as the production of metabolites and the bacterial composition of the microbiota. *Drosophila melanogaster* provides a well-suited model organism to unravel these interactions in the context of nutrigenomics as it combines several advantages including an affordable maintenance, a short generation time, a high fecundity, a relatively short life expectancy, a well-characterized genome, and the availability of several mutant fly lines. Furthermore, it hosts a mammalian-like intestinal system with a clear microbiota and a fat body resembling the adipose tissue with liver-equivalent oenocytes, supporting the fly as an excellent model organism not only in nutrigenomics but also in nutritional research. Experimental approaches that are essentially needed in nutrigenomic research, including several sequencing technologies, have already been established in the fruit fly. However, studies investigating the interaction of a specific diet and/or dietary compounds in the fly are currently very limited.

The present review provides an overview of the fly’s morphology including the intestinal microbiome and antimicrobial peptides as modulators of the immune system. Additionally, it summarizes nutrigenomic approaches in the fruit fly helping to elucidate host-genome interactions with the nutritional environment in the model organism *Drosophila melanogaster*.

## Background

Nutrigenomics defines the nutrient-gene interactions in a host and at present includes not only nutrient-gene interactions but also nutrient-epigenetic, nutrient-proteomic, and nutrient-metabolomic interactions as well as host-diet-microbiome interactions [1]. In this sense, nutrigenomic research is located on the intersection between diet, health, and genomics [2, 3].

*Drosophila melanogaster* is a model organism essentially applied in genetic research that brings promising advantages into studying preclinical nutrigenomics. Its evolutionary biology significantly contributes to the understanding of gene expression and development in humans, as its genome conserves approximately 60% of genes that are related with DNA mutations, amplifications, or deletions in a diverse set of human diseases [4, 5]. Its genome encodes ca. 18,000 genes located on four homologous pairs of chromosomes, while only three of them hold the main part of the genome. Flies and mammalian species normally share about 40% of the nucleotide and protein sequences in their homologs; in some conserved functional domains, it can be more than 90% [6, 7]. Chromosomal deletions and mutations have been generated for the production of *Drosophila melanogaster* mutants, targeting more than 80% of its genome [8].

Beside its well-characterized genome and the good availability of mutant and transgenic flies, other advantages including a rapid life cycle (12 days for the succession of egg, maggot, pupa, and imago), a short life span (around 70–80 days), a small size (possibility of breeding hundreds of individuals in small bottles), and a relatively easy generation of mutant animals in comparison to other organisms make *Drosophila melanogaster* an excellent model organism in nutrigenomic research.

Particularly, due to the presence of a fat body with adipocytes and conserved metabolic pathways involved in fat metabolism and insulin signaling, *Drosophila melanogaster* has been extensively used to investigate obesity-associated diseases, including cardiovascular dysfunction or cancer [9–11]. Changes in triglyceride levels and lipid storage induced by the intake of high-fat and high-sugar diets have been related with genetic variations in both genes of the insulin/insulin-like growth factor signaling (IIS) and the target of rapamycin (TOR) signaling pathway [12, 13].

The fruit fly also resembles a good model to study different tissues or organs due to its mammalian-like anatomy and equivalent functions. The present review provides information on the fruit fly’s morphology and anatomy with a special focus on the gastrointestinal system and the gut microbiota, key facts in nutrigenomics studies. Additionally, it gives insights into the different methods applied in nutrigenomics and their utilization in *Drosophila melanogaster*.

### *Drosophila melanogaster*—morphology

*Drosophila melanogaster* presents different morphology traits as a product of natural selection. These differences are generally associated with gene mutations referring to single phenotypes [14]. Mutation markers indicating differences in bristles, wings, appendages, eye shapes, and colors and body sizes have been collected by FlyBase (www.flybase.org), providing accurate information about its location in the chromosomes. Environmental factors, such as nutrition, temperature, or crowding, have been reported to be responsible for morphological traits, in particular, body size variations, and have been connected to quantitative trait loci (QTL) mapping on the third chromosome while no QTLs or QTLs with minor effects on these factors have been detected in the other major chromosomes [14]. The time for fly development, also known as the fruit fly life cycle, varies within different environmental conditions. Generally, the development of new flies takes around 10 days at 25 °C with four developmental stages: the embryo, larvae (three different stages), pupal stage, and imago stage. The adult flies reach sexual maturity 2–4 days after eclosion.

According to a typical insect morphology, the adult fruit fly body is divided into three parts: head, thorax, and abdomen. In the head, there are several sensory organs, noting the compound eyes, containing primary pigments being characteristic for different mutants, and the proboscis, representing the gustatory organ for food detection, taste, and intake, which could be extended and retracted and pumps the food into the gut. The thorax is divided into three sections: prothorax (anterior) with one pair of legs, mesothorax (middle) with one pair of legs and one pair of wings, and metathorax (posterior) with one pair of legs and one pair of halters (modified wings). Females and males can be easily differentiated by morphological attributes, especially, females are generally bigger and possess an abdomen that has a pointed tip whereas males show a rounded abdomen with black pigmentation in the posterior segment with an epandrium (male external genitalia) [15].

The anatomy of the fly includes organ systems with equivalent functions to mammalian organisms, including the brain, peripheral nervous system, heart, trachea system (similar to the lung), esophagus, Malpighian tubules (similar to the kidneys), fat body with oenocytes (combining the functions of adipose tissue and the liver), gut, and gonads [16]. The fly brain possesses more than 100,000 neurons and exhibits important functions in a similar way as in the mammalian central nervous system, including circadian rhythms, sleep, learning, memory, courtship, feeding, aggression, grooming, and flight navigation. Therefore, this model organism offers the possibility to investigate feeding-associated behaviors by analyzing metabolic changes in conjunction with neuroendocrine and neuromodulatory states and underlying molecular mechanisms [17]. It has been documented that flies react to various dietary compounds or drugs within their central nervous system in a similar way as observed in mammalian systems [6].

Regarding the significant importance of the digestive tract in the context of nutrition research, the present review provides detailed information on the digestive tract including its microbiota. *Drosophila melanogaster’s* alimentary canal consists of a simple epithelium which encompasses visceral muscles, trachea, and nerves [18]. Depending on their position along the gut length, these different cell types differ in their arrangement and functions which may vary on their different developmental origins [18]. The intestinal epithelium of the fruit fly consists of a monolayer of four different types of cells: intestinal stem cells (ISC), absorptive enterocytes (EC), secretory enteroendocrine (EE) cells, and enteroblasts (EB). The last may differentiate either into an EC or an EE depending on the different signals present in specific parts of the fly’s digestive tract [19]. Under normal physiological conditions, the ISC proliferate and differentiate in a rate that maintains a correct intestinal barrier function [20]. During aging, proliferation and differentiation of these ISC may be impaired resulting in epithelial dysplasia [21]. A loss of ISC, a disturbed epithelial turnover, and an impaired epithelial ultrastructure have been suggested to cause a decrease in *Drosophila melanogaster* life span following the uptake of the probiotic strain *Lactobacillus plantarum* [20]. These results are in contrast to other studies showing beneficial effects of *L. plantarum* especially in the context of developmental rates and ISC proliferation in young *Drosophila melanogaster* [22–26]. This suggests that the effects of health-promoting gut microbes may also depend on various factors including age and genotype as well as the applied probiotic strains and the diet [26].

The fly’s alimentary canal is roughly divided into foregut, midgut, and hindgut [27]; while the foregut is of ectodermal origin, the midgut and the hindgut are—as all other organs of the fly—of endodermal origin [28]. Specifically, the foregut consists of the mouth, the pharynx, the esophagus, and the crop [29], an organ for the storage and mixing of food, as well as for the detoxification. The foregut is connected with the midgut by the cardia, a sphincter that controls the food passage [27]. The midgut is the central part of the digestion as digestive enzymes are excreted and nutrients are absorbed [30]. Historically, the midgut has been further divided into the anterior, middle, and posterior part, while it has been recently classified into six different anatomical regions (R0–R5) exhibiting specific metabolic and digestive functions [30]. Within the midgut, a region with a pH of < 4.0 exists, indicating that the so-called copper cells secrete acid—like the parietal cells in the mammalian stomach—which in consequence helps to digest proteins [18] and supports the permanent colonization of the alimentary tract with commensal bacteria [21]. Similar to the mammalian mucus layer, the midgut of the fly is lined by a peritrophic matrix (PM) that is produced by the crop and is composed of glycoproteins and chitin, potentially protecting the midgut epithelium from harmful particles and microbes [29].

### *Drosophila melanogaster*—microbiota

In humans, the gastrointestinal tract is populated by a multiplicity of microorganisms including more than 500 different bacterial species. In the present context, the so-called microbiota refers to the commensal bacteria present in the colon [31]. In healthy human subjects, the microbiota shows a distinguished composition that consists of five phyla: mainly *Bacteroidetes* (*Bacteroides* ssp.) and *Firmicutes* (*Lactobacillus* spp.), and also *Actinobacteria* (*Bifidobacterium* ssp.), *Proteobacteria* (*Escherichia, Helicobacter*), and *Verrucomicrobia* (*Akkermansia* spp.) [32, 33]. This microbiota composition is vulnerable during childhood and advanced age and rather stable during adulthood [33]. Various studies have reported a high microbiota diversity between subjects suggesting an association with different diets and obesity and consequently in energy homeostasis [32].

Taking advantage of the sophisticated genetic tools available in the fruit fly *Drosophila melanogaster*, its complex gastrointestinal system and the presence of a clear microbiota, it would be a predestined model to unravel host-microbiota interactions related to nutrition. The gut of *Drosophila melanogaster* hosts a limited number of commensal gut bacteria ranging from 3 to 30 species, including *Lactobacillus plantarum* as the most prevalent, *Acetobacter pomorum*, *A. tropicalis*, *L. frucitvorans*, and *L. brevis* [34, 35]. Interestingly, populations of *Lactobacillus* species are common to both fly midguts and animal small intestines [36] and have been associated with several biological functions in *Drosophila melanogaster*, including larval growth, food uptake, and protection from malnutrition or oxidative stress, similar to health-promoting properties of *Lactobacillus* in mammals [37]. Laboratory fly stocks are associated with a relatively low number of taxa (about 1–13 OTUs define around 97–99% of identity, depending on the study), while most bacteria refer to two genera: *Acetobacter* and *Lactobacillus* [38]. Large changes in both microbial load and composition of bacterial species in the *Drosophila* intestinal microbiota are —similar to humans—more closely associated with the animal’s gut morphology, epithelial architecture, and health status than with its chronological age [23]. It is not astonishing that some studies have reported different bacterial compositions in the gut of wild and laboratory strains of *Drosophila melanogaster*, supporting the assumption that the microbiota mainly corresponds to bacteria growing on the ingested foods and rather needs a permanent and repeated ingestion through the diet to permanently colonize the fly’s intestine [39, 40]. Similarly, Pais et al. (2018) reported that laboratory stocks (w^1118^) host mainly two bacterial species in their gut corresponding to *Acetobacter* OTU2753 and *Lactobacillus* OTU1865, which, however, cannot persist in the gut without a reinfection via their foods. Interestingly, in wild-caught fruit flies, 35 different OTUs, corresponding to *Enterobacteriaceae, Acetobacteriaceae* (mainly *Acetobacter* and *Gluconobacter* species), *Leuconostocaceae*, and *Bacillaceae,* were identified as the most prevalent families, partly containing bacterial strains that are able to stably colonize the fly gut, such as *L. pseudomesenteroides*, *A. cibinongensis*, and *A. thailandicus* [41]. Therefore, a further characterization of the host’s interaction with persistent gut-colonizing bacteria would contribute to a better understanding in the context of *Drosophila*-microbe interactions. Nevertheless, several studies have demonstrated a significant impact of *Drosophila* gut commensal microbes on host-signaling pathways, metabolic capacities, development, locomotion, immune response, intestinal functionality, and aging, demonstrating that an excessive bacterial growth or dysbiosis promotes the organism’s death [42].

Sterile or axenic fly strains (reared under germfree conditions) may be generated either by applying low doses of streptomycin to the diet or by performing egg dechorionation [43]. To obtain flies with a defined microbial community (gnotobiotic flies), flies will either be exposed to correspondingly inoculated sterile diets or embryos will encounter microbial species of interest [44]. In an experiment using axenic and gnotobiotic flies, Dobson et al. [45] compared the co-expression of specific and functionally related genes associated with growth, metabolism, and neurophysiological regulators (such as the components of the IIS and TOR pathways), showing an upregulation of these genes in the presence of the microbiota, and consequently its influence on the host transcriptome [45]. A recent publication demonstrated that the elimination of the microbiota altered the expression of immune response-associated genes, as well as genes connected with oxidative stress and general detoxification, in the head of young adult *Drosophila melanogaster* [46].

### Nutrigenomic approaches in *Drosophila melanogaster*

**Fig. 1:**
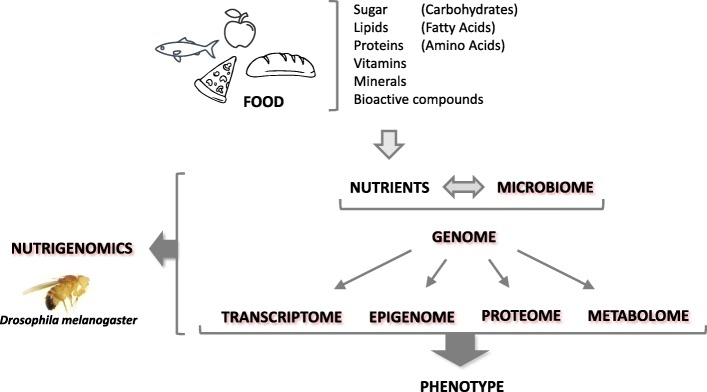
Overview of the nutrigenomics approach in the model organism *Drosophila melanogaster.* An organism ingests complex foods which are degraded into nutrients that interact with the microbiome. This in consequence affects the genome, involving the transcriptome, the epigenome, the proteome, and the metabolome, resulting in the organism’s phenotype. The fruit fly can be used as a model organism in nutrigenomics, as changes in the microbiome, transcriptome, epigenome, proteome, and metabolome due to an interaction with the nutritional environment are detectable and evaluable by several methods (pictograms used are from *vecteezy.com*)

As mentioned earlier, nutrigenomics refers not only to gene-nutrient interactions but also to nutrient-epigenetic, nutrient-proteomic, nutrient-metabolomic, and nutrient-microbiome interactions (Fig. 1).

Diverse transcriptomic tools may be used in nutrigenomics research in *Drosophila melanogaster* including microarrays, to deliver information on changes in the mRNA expression following the dietary intake of a specific nutrient [7], and RNA sequencing [10] and next-generation sequencing (NGS) technologies [47], to analyze regions of interest in the genome, providing promising results and solutions to nutrigenomics studies by identifying new mutations in inbred fly strains. In addition, studies of QTL [48], representing a genome region that causes a significant variation in a quantitative trait, may be used in identifying signaling pathways involved in the metabolism of specific nutrients. An important goal achieved in *Drosophila* genetics research is the generation of an RNAi knockdown fly line collection by the Vienna Drosophila Research Centre, targeting around 90% of the whole fly genome and being accessible for the research community [6]. Until then, large-scale RNAi screens of gene function have been mainly performed in *Caenorhabditis elegans*, although it exhibits systemic RNAi for which reason the gene interference cannot be referred to a specific cell type [49]. As RNAi of *Drosophila melanogaster* is cell autonomous, it can be activated by inserting a transgenic long double-stranded “hairpin” RNA [49]. By combining this tool with the GAL4/UAS system in *Drosophila*, it offers the possibility to inactivate the expression of a specific gene in various different cell types helping to generate conditional transgenic fly models [50]. This makes it easier to study the overexpression or the misexpression of fly homologous genes and proteins, helping to establish fly models to study human diseases.

#### Genomics

The genome refers to the genetic material of an organism consisting of DNA. Genes (coding regions of the DNA) and non-coding regions of the DNA, mitochondrial DNA as well as chloroplast DNA, are parts of the genome [51]. The *Drosophila* genome has a size of 180 Mb and is packed into four pairs of chromosomes, and the genome sequence has been known for nearly 20 years [52]. Each of the large chromosomes contains a DNA molecule with 5 cm in length that has to fit into a nucleus with a diameter of ca. 5 *μ*m. This indicates that the chromosomes need to be condensed several thousand times to fit perfectly into the small nucleus which is mediated by chromatin folding. During the last decades, it has become obvious that this DNA organization essentially contributes to the regulation of the gene expression which is referred to as epigenetic regulation [52].

#### Transcriptomics

The transcriptome refers to all messenger RNAs present in one cell or a population of cells at a defined time [53]. The analysis of the transcriptome has been mainly dominated by microarray analysis provided by different companies, including Affymetrix, Agilent Technologies, and Illumina. Recently, these analyses have been based on the RNAseq technology, defined as transcriptome profiling using NGS. It is stated that this methodology offers—compared to microarrays—the advantage of the detection of lower abundant and wider ranges of transcripts [54]. By comparing the intake of two different obesogenic diets, RNAseq analysis from *Drosophila* heads revealed significant differences in the transcriptome. While genes associated with immunity, metabolism, and hemocyanin have been mainly affected in flies fed with a high-fat diet, genes connected with cell cycle checkpoint kinases (CHK), cell cycle activity, and DNA binding and transcription have been upregulated in flies receiving a high-sugar diet [10]. In a recent study by Azuma and colleagues [55], plant bioactives have been applied to detect antiobesogenic effects in a fly model of obesity. RNAseq analysis has been performed to detect differentially regulated genes in male and female flies fed with a coconut-oil-supplemented high-fat diet, either in the presence or in the absence of quercetin glycosides (QG) or epigallocatechin gallate (EGCG). This is—as far as we know—one of the first publications presenting lists of differentially regulated genes in obese flies using RNAseq data analysis. These results have been supported by functional analysis showing lower triglyceride levels in flies under QG or EGCG supplementation. This study, as well as our own experiment demonstrating a clear visual separation of the fly’s transcriptome following a dietary supplementation of the secondary bile acid lithocholic acid (LCA), pushes the fruit fly as an excellent model organism in nutrition research and, specifically, in the context of transcriptomic analysis. Gene set enrichment analysis has shown a downregulation of TOR, metabolism, Wnt, p53, and immune processes, whereas genes associated with the cell cycle have been increased following dietary LCA treatment [56]. An earlier study by Ye and colleagues [7] performed transcriptomic analysis by using the microarray technology. Preliminary results have been generated in flies being exposed to different energy sources in their diets, including sucrose as a control, palmitic acid, soy, and beef. Changes in the gene expression levels of ca. 2–3% within the ca. 18,000 genes have been observed following the intake of the different diets [7]. Additionally, in *Drosophila* larvae, a starvation of amino acids changed the transcriptome, especially metabolism-associated genes, mainly involved in the TOR pathway [57].

#### Epigenetics

The term epigenetics defines heritable phenotype alterations which are not mediated by a change in the DNA sequence. Epigenetic changes are mediated by histone modifications, DNA methylation, and microRNA expressions [58]. The epigenome changes within the cells and is more dynamic compared to the genome [59]. It has been documented that our diet is able to induce epigenetic alterations that, in consequence, affect biomarkers of metabolic modulations in different model organisms as well as in human subjects. A very famous example of epigenetic effects due to dietary changes are humans that survived the so-called Dutch hunger winter in 1944 [60]. Several years later, researchers were able to detect changes in different metabolic markers in their offspring, such as the glucose tolerance [61], which resulted from a change in the methylation pattern of specific genes due to a limited availability of calories during the gestational period [62, 63]. To detect epigenetic changes in a biological sample, MethyLight technology, pyrosequencing, chromatin immunoprecipitation-on-chip (ChIP-on-chip), and quantitative methylation-specific polymerase chain reaction (QMSP) followed by pyrosequencing can be applied [59]. All methods use the sodium bisulfite treatment as the compound reacts with unmethylated cytosine and converts it into uracil, which helps to deliver information on DNA methylation via PCR technology [59]. The detection of changes in microRNA expression is mainly performed by gene-chip microarray technology (Affymetrix), while histone modifications are detected by applying specific monoclonal antibodies against histone modifications or by a ChIP-seq assay followed by NGS [59].

Studying diet-related effects on epigenetic mechanisms in fruit flies has just recently started [64, 65]. The administration of diets with a varying macronutrient composition shows persistent changes of genes associated with epigenetic mechanisms over generations [64]. A study by Lian and co-workers [65] looked into the DNA methylation pattern of flies reared under dietary restriction. Unexpectedly, the methylome of these flies exhibited only minor changes which may be due to the relatively young age (7 days) at the sampling day as changes in the life span due to dietary restriction usually occur at a later time point [66]. Further research looking into DNA methylation pattern in flies under dietary restriction at an older age would therefore provide more valuable data regarding epigenetic modulations. Another possibility to check epigenetic changes is to study chromatin remodeling. In this regard, Sebald and colleagues demonstrated a central role of the chromatin remodeling factor CHD1 on a healthy microbiome composition in the fruit fly [67], which indirectly indicates an effect of the diet, as it is the most prominent factor affecting the intestinal commensal bacteria [68, 69]. This study exemplified the fruit fly as an upcoming model organism in epigenetic research, helping to elucidate diet-dependent effects on the epigenome. In the context of epigenetic research, the fruit fly offers the advantage to investigate epigenetic effects throughout different generations during a relatively short period of time.

Other molecules that epigenetically modify gene expression are microRNAs (miRNA), small non-coding RNAs with a length of 17–25 nucleotides, normally inhibiting gene expression. Their main type of action is via (a) an inhibited translation and/or (b) by inducing the degradation of the mRNAs, known to be centrally involved in the epigenetic regulation of gene expression [70]. MicroRNAs play a central role in cellular processes such as proliferation, differentiation, and apoptosis, which are known pathways affected in the development of chronic diseases including cancer [71]. Studies have shown that especially plant bioactives are able to affect miRNA expression which may partly explain their health-promoting properties documented in the development of various chronic inflammatory diseases [72–74]. Initial experiments identified lin-4 as the first miRNA being essential for the normal development of *Ceanorhabditis elegans* [75]. *Drosophila melanogaster* has also been successfully used to generate essential information on effects of miRNA, by establishing the Flp-FRT and GAL4-UAS systems, allowing to knock-in or knock-out specific miRNAs with particular functions in the fly [71]. The state-of-the-art technology CRISPR/Cas9 has been recently established in the context of miRNA research as miR-219 and miR-315 have been successfully knocked down in *Drosophila melanogaster* [76]. This fact points towards an important input in elucidating miRNA-based processes [71]. In addition, it offers the possibility to use the fruit fly as a model organism to elucidate health-promoting or health-declining effects of different macronutrients and/or specific food components potentially related to miRNA modulation.

#### Proteomics

The proteome is defined as the protein complement that is present in a cell, an organ, or an organism at a given time [54, 77]. As proteins present the functional part of genes and the mRNA information, the proteome accounts for the organism’s phenotype [77]. Data regarding the proteome of *Drosophila melanogaster* in connection to different diets and/or dietary compounds are currently very limited. Li and co-workers demonstrated a change in the midgut proteome of the fruit fly receiving the Bowman-Birk protease inhibitor via their diet [78]. In comparison to control diet-fed animals, the proteomic analysis in fly larvae exposed to this inhibitor showed an impaired expression of proteins associated with protein degradation and transport, as well as fatty acid catabolism [78]. Another study investigated the effect of dietary ethanol on the proteome of fruit flies. Culwell and colleagues have detected relatively stable proteomes following the treatment with 10% ethanol compared to control-fed flies [79]. Admittedly, the authors have only focused on short-term effects of the applied compound with the intention to confirm the so-called Hamburger effect, which has been suggested for human proteomes following the consumption of one single hamburger [80].

In addition, antimicrobial peptides, including metchnikowin, diptericin, attacins, cecropinA1, and drosocin, have been widely used as biomarkers for the *Drosophila melanogaster* immune system, playing a crucial role in the defense mechanisms, the stem cell proliferation, and the regulation of the gut microbiota in mammals [47]. The identification and quantification of different antimicrobial peptides by mass spectrometry technologies and gel electrophoresis, as well as their expression levels using qRT-PCR and NGS, may be evaluated to get information on the health status and especially on the immune status of *Drosophila melanogaster* receiving different diets or supplements such as bioactive compounds. Altered anti-microbial peptide levels have been related to an impaired proliferation of ISC and intestinal bacterial loads. In particular, an increased expression of the antimicrobial peptides drosocin and cecropin A1 in the intestine has been connected with a prolonged life span of flies [81]. This increased expression of drosocin and cecropin A1 is associated with a lower activation of the classical immune pathways in the midgut of these flies, such as the immune deficiency (IMD) and Janus kinase-signal transducers and activators of transcription (JAK-STAT) pathway, as well as with lower activities of c-Jun N-terminal kinase (JNK) and epidermal growth factor (EGF) which points towards a better regeneration and maintenance of ISC and an alleviated stress response [81]. In a recent publication, Hanson and colleagues [82] used flies lacking all 14 antimicrobial peptides, that have been systematically tested for their effects on Gram-positive and Gram-negative bacteria and fungi. The *Drosophila* antimicrobial peptides mainly affect Gram-negative bacteria and represent rather effectors than regulators of the innate immune system in the fruit fly [82]. Effectors are built in an immune reaction with an antigen while regulators mainly repress ongoing immune reactions.

Although only limited information of diets and/or specific nutrients/nutritional factors on the fly proteome are currently available, the fruit fly could be a suitable model organism to unravel effects of specific diets/nutrients/bioactive ingredients on the protein expression. Methods to detect alterations of the proteome include (a) methods to separate the proteins and (b) methods to identify and characterize the proteins. Extractions, precipitations, chromatography, electrophoresis, and centrifugation can be applied to separate the proteins, while mass spectrometry, nuclear magnetic resonance (NMR) spectroscopy, and immune labeling can be used for protein identification and characterization.

#### Microbiomics

The gut microbiota in the fruit fly can be isolated after the dissection of the gut or from the whole fly [23]. By using the whole fly, usually, the surface is disinfected by ethanol in order to remove external bacteria. In addition, a non-invasive approach can be applied by collecting and analyzing fecal spots that have been deposited by the flies during a defined period [83]. This offers the advantage of analyzing microbiota dynamics in the same cohort at several time points, like throughout a life span experiment or nutritional interventions. As far as we know, there are only a few studies available in *Drosophila melanogaster* that have analyzed the microbiota composition after applying a specific diet or a specific dietary compound. Recently, Erkosar et al. [84] have demonstrated a drastic effect on the abundance and the α-diversity of the intestinal microbiota in fruit flies following the intake of specific nutrients. The authors observed a 100-fold induction in the total abundance of bacterial members of the *Drosophila* microbiota by increasing dietary yeast from 4 to 27% in the fly food [84]. In another study, the bioactive compound ursolic acid has been added to the fruit fly diet, which resulted in a shift of the gut microbial composition mainly affecting *Lactobacillus*, *Acetobacter*, and *Actinobacteria*, potentially related to an increased life span and climbing activity, as well as an overexpression of the *Spargel* gene (PPARγ-coactivator 1 α (PGC-1α) homolog) in the male fruit fly [85].

The bacterial diversity and alterations in microbiota dynamics in the fruit fly can be analyzed by using 16S rRNA gene sequencing by different methodologies, such as a qPCR approach with species-specific oligonucleotide primer pairs [83], deep gene sequencing approaches using 454 sequencing [38] or whole-genome shotgun sequencing [86], and high-sensitive NanoString nCounter technology for targeted RNA, DNA, or proteins [87]. Initial data also point towards the use of flow cytometric microbiome analysis as an easy-to-use and cost-effective method to unravel effects on the *Drosophila* microbiota. Although this method does not deliver direct phylogenetic information, it provides information about relative subcommunity abundance and absolute cell numbers at-line through distinct light scatter and fluorescence properties [88]. Staats and colleagues have already used a flow cytometry-based analysis together with the sequencing of the V1-V2 regions of the 16S rRNA to detect changes in the microbiome of *Drosophila melanogaster* following the intake of the plant bioactive ursolic acid [85].

*Drosophila melanogaster* has also been demonstrated to be a successful in vivo model system to elucidate the mechanisms of probiotic organisms in the human microbiota (i.e., “beneficial” bacterial species such as *Bifidobacterium bifidum*) by pathogen inhibition [39, 89]. Recently, the probiotic strain *Lactobacillus fermentum* NCIMB 5221 and its metabolite ferulic acid have been added to the *Drosophila melanogaster* larvae medium, targeting the TOR and IIS signaling pathways as well as the larvae’s metabolism resulting in an acceleration of its developmental growth [90]. Therefore, elucidating potential molecular pathways of probiotics or its corresponding metabolites by using the fruit fly as a model organism would help to improve therapies for human diseases related to the energy metabolism, such as obesity and diabetes.

#### Metabolomics

Metabolomics is referred to a systematic study of detectable small molecules deriving from specific cellular processes in an organism [54]. Metabolomics studies could provide information on the effects of dietary compounds, and their health consequences, on an organism’s metabolism. The interpretation of the results is relatively difficult as these metabolites may derive from at least three different sources: (1) from the diet (nutrients/bioactive compounds), (2) from incorporated environmental xenobiotics, and (3) metabolic signals generated by the commensal gut bacteria (microbiota) [54]. The main technologies applied in metabolomics research are mass spectrometry and NMR spectroscopy, both having advantages and disadvantages [59].

*Drosophila melanogaster* is a well-known model in the context of metabolomics research [91, 92]. However, studies in the context of diet-metabolome interaction are currently very limited. An and Fukusaki [92] provided a list with studies in the fruit fly using metabolomics approaches. Heinrichsen and co-workers [93] analyzed the metabolome of *Drosophila melanogaster* that received a high-fat diet (HFD). In this study, the metabolome of HFD-fed flies showed changes in the metabolism of fatty acids, amino acids, and carbohydrates compared to control diet-fed flies. In another experiment, fruit flies under dietary restriction exhibited different metabolic profiles compared to the corresponding control flies, suggesting a central role of dietary restriction in the prevention of age-associated pathologies [94].

## Conclusion and outlook

*Drosophila melanogaster* can be established as a well-suited model system in nutrigenomics research due to the fact that it is one of the best-characterized model organisms in genetic research. The fruit fly also offers the possibility to study nutrition-related effects on the genome as the main methods and techniques required are already established. This model organism is also useful for host-microbiota interactions, as *Drosophila melanogaster* hosts only a small number of bacterial populations in its gut including species also present in the human microbiota. In general, animal welfare ethical review boards do not have to approve experimental settings applying the fruit fly, providing an essential advantage compared to, for example, laboratory rodents [95]. The ideal use of the fruit fly in nutrition and nutrigenomics research would be the establishment of a screening platform delivering essential information on host-genomic interactions. It would assist to discover and validate primary small molecules and narrow it down to the most potential candidates, which may then be tested in rodents and eventually in humans [6]. In this context, *Drosophila melanogaster* offers additional advantages compared to other research organism models, including a short generation time, a high fecundity, a small genome size, the presence of a high number of genes and conserved metabolic signaling pathways connected with human diseases, a good availability of mutant fly strains, and a relatively cheap maintenance [6]. The fruit fly may also be applied to elucidate the effects of different diets and bioactive compounds, as well as different microbial strains, on the immune system by evaluating the anti-microbial peptide expression in connection with systemic inflammation and gut homeostasis [95, 96]. However, to completely understand the link between genotype, microbiota, and diseases, major obstacles such as the microbial diversity and the genetic complexity of the immune system need to be taken into consideration. The fruit fly gut microbiota can be isolated, cultured, and engineered in a relatively easy way, offering also a convenient model system to analyze microbiota-associated diseases, including metabolic, neurological, and immunological disorders [16, 97]. The use of *Drosophila melanogaster* in epigenetics has only been recently introduced and, therefore, needs more research regarding the underlying mechanisms involved in genome stability and regulation [98, 99]. Additionally, a *Drosophila melanogaster* model exhibiting specific metabolic deficiencies may be applied to deliver information on dietary and/or pharmaceutic interventions contributing to a personalized nutrition approach prior to a costly testing in a human trial [100]. As recently suggested by Lüersen and colleagues [101], standardized interlaboratory models and protocols for *Drosophila melanogaster* maintenance are essentially needed which also apply to nutrigenomics research. Finally, this will contribute (a) to the validation of nutrition-based experiments and (b) to a better comparability of nutrition-related studies.

## Abbreviations

CHD1: Chromatin remodeling factor 1
ChIP: Chromatin immunoprecipitation
CHK: Cell cycle checkpoint kinases
EB: Enteroblast
EC: Enterocyte
EE: Enteroendocrine cell
EGCG: Epigallocatechin gallate
HFD: High-fat diet
IIS: Insulin/insulin-like growth factor signaling
IMD: Immune deficiency
ISC: Intestinal stem cell
JAK-STAT: Janus kinase-signal transducers and activators of transcription
JNK: c-Jun N-terminal kinase
LCA: Lithocholic acid
miRNA: microRNA
NGS: Next-generation sequencing
NMR: Nuclear magnetic resonance
OTU: Operational taxonomic unit
PGC1α: PPARγ co-activator 1α
PM: Peritrophic matrix
QC: Quercetin glycoside
QTL: Quantitative trait loci
rRNA: Ribosomal RNA
TOR: Target of rapamycin
UAS: Upstream activation sequence
